# Tunable recombinant protein expression in *E*. *coli*: promoter systems and genetic constraints

**DOI:** 10.1007/s00253-016-8045-z

**Published:** 2016-12-21

**Authors:** Lukas Marschall, Patrick Sagmeister, Christoph Herwig

**Affiliations:** 1 Institute of Chemical Engineering, Research Area Biochemical Engineering, Vienna University of Technology, Vienna, Austria; 2 Exputec GmbH, Vienna, Austria; 3 Christian Doppler Laboratory for Mechanistic and Physiological Methods for Improved Bioprocesses, Vienna University of Technology, Gumpendorferstrasse 1a/166-4, A-1060 Vienna, Austria

**Keywords:** All-or-none induction, *E. coli*, Promoter, Transcription, Tunable

## Abstract

Tuning of transcription is a promising strategy to overcome challenges associated with a non-suitable expression rate like outgrowth of segregants, inclusion body formation, metabolic burden and inefficient translocation. By adjusting the expression rate—even on line—to purposeful levels higher product titres and more cost-efficient production processes can be achieved by enabling culture long-term stability and constant product quality. Some tunable systems are registered for patents or already commercially available. Within this contribution, we discuss the induction mechanisms of various *Escherichia coli* inherent promoter systems with respect to their tunability and review studies using these systems for expression tuning. According to the current level of knowledge, some promoter systems were successfully used for expression tuning, and in some cases, analytical evidence on single-cell level is still pending. However, only a few studies using tunable strains apply a suitable process control strategy. So far, expression tuning has only gathered little attention, but we anticipate that expression tuning harbours great potential for enabling and optimizing the production of a broad spectrum of products in *E*. *coli*.

## Introduction

Tuning of transcription is a promising technology for efficient recombinant protein production in *Escherichia coli*. Many challenges like inclusion body formation (Baig et al. 2014; Hartley and Kane 1991), metabolic burden (Bentley et al. 2009; Bienick et al. 2014; Glick 1995; Mairhofer et al. 2013), inefficient translocation (Baneyx and Mujacic 2004) and outgrowth of segregants (Beisel and Afroz 2016) associated with a non-suitable expression rate can be addressed by adjusting the expression rate to purposeful levels (Marschall et al. 2015). This can either be achieved by using a low producing mutant strain or a host harbouring, a tunable promoter system, which offers the possibility to vary the protein expression on line on cellular level. In contrast to the use of various mutants with diverse expression strengths, a tunable strain opens up new possibilities. Continuous processing could benefit from the on line controllability of the transcription rate resembling an adjustment knob to manoeuvre an “out of specs” process back into the design space and enable long-term production periods (Marschall et al. 2015). A handful of contributions deals with that promising technology, two claimed-to-be tunable strains are commercially available (Tuner™, Novagen and Lemo21 DE3, New England Biolabs) (Schlegel et al. 2012; Turner et al. 2005), and one is registered for patent approval (Jay D. Keasling 2012).

Defining transcription tuning as the purposeful adjustment of the recombinant gene transcription rate on cellular level, we recently reviewed genetic and process technological aspects of transcription tuning (Marschall et al. 2015), identified certain prerequisites for tunable promoter systems and proposed a roadmap for development of industrial tunable expression systems. In order to be tunable on a cellular level, a tunable promoter system must not exhibit all-or-none induction, i.e. the formation of subpopulations of producing and non-producing cells, but has to respond to the inducer in a dose-dependent manner (Marschall et al. 2015). Some promoter systems, which in their native form respond in an all-or-none fashion, can be rendered tunable by genetic alterations or process technological means. Therefore, the induction mechanisms and kinetics of the used promoter systems as well as process technological aspects need to be considered. Though several *E*. *coli* promoter systems have been investigated in various studies, a comprehensible review of *E*. *coli* promoter systems with respect to their tunability is still missing.

While our first review aimed more at process technological solutions for expression tuning and its implementation in an industrial context, within this contribution, we would like to fill the previously described gap and review induction mechanisms of common *E*. *coli* inherent promoter systems with respect to their tunability and discuss genetic and process technological solutions to achieve tunability for each system. A lot of different promoter systems for recombinant protein expression in *E*. *coli* are available and applied in research and industry (Balzer et al. 2013; Brautaset et al. 2009). However, within this article, we would like to focus on *E*. *coli* inherent promoter systems. In addition to that, we discuss reports that claim to apply transcription tuning and set them into context with the current knowledge on their induction mechanisms and kinetics.

## Promoter systems for tunable recombinant protein expression

In the following section, promoter systems that are reported to feature tunability are reviewed. For each system, a short overview of general characteristics is given. Subsequently, its functionality is reviewed and discussed with respect to its tunability. The systems discussed in this section are outlined in Table 1.

**Table 1:** Quick recap in literature reported systems with respect to tunability. “Proof of tunability” refers to whether or not tunability on cellular level was demonstrated for the respective system

Promoter	Genotype	Add. plasmid	Inducer	Proof of tunability	Comments	Reference
**p**_**lac**_	Complete	–	Lactose	No	According to lactose p_lac_ induction mechanistic (Afroz et al. 2014b), probably tunable	(Striedner et al. 2003)
	Complete	–	IPTG/TMG	No	According to IPTG p_lac_ induction mechanistic (Afroz et al. 2014b), probably not tunable	(Striedner et al. 2003)
	*lacY-*	–	IPTG/TMG	Yes		Tuner™, (Turner et al. 2005)
	*lacY- lacA-*	–	IPTG/TMG	Yes		
**p**_**araBAD**_	Complete	–	Arabinose mixed feed	Yes		(Sagmeister et al. 2013b)
	*araE- araFGH-*	*araE* under control of different promoter	Arabinose	Yes		(Khlebnikov et al. 2002)
	*araBD-*	–	Arabinose	No	According to arabinose p_araBAD_ induction mechanistic (Afroz et al. 2014b), probably not tunable	(Sommer et al. 2010)
	*araBAD-*	–	Arabinose	No		
**p**_**rhaBAD**_	Complete	pLemo	Rhamnose/IPTG	/		(Wagner et al. 2008)
**p**_**rhaT**_	Complete	–	Rhamnose	/		(Giacalone et al. 2006)
**p**_**proU**_	Complete	–	NaCl	No	According to NaCl p_roU_ induction mechanistic (Lucht and Bremer 1994), probably tunable	(Herbst et al. 1994)
**p**_**prpB**_	Complete	–	Propionate	Yes		(Lee and Keasling 2005)

## Plac system

### General characteristics of the plac system

The p_lac_ promoter can either be induced by its natural inducer lactose (allolactose) and by gratuitous inducers like isopropyl β-D-1-thiogalactopyranoside (IPTG) and thiomethyl-β-D-galactoside (TMG) (Herzenberg 1959). Like lactose, TMG and IPTG are both recognized by lactose permease (LacY) and can therefore enter the cell membrane either by active or diffusive transport or a combination of both (Fernández-Castané et al. 2012; Hansen et al. 1998; Maloney and Rotman 1973; Marbach and Bettenbrock 2012). The intracellular inducer concentration can also decrease in course of the process: lactose can be metabolized by *E*. *coli* using its inherent sugar uptake pathways. TMG and IPTG cannot be metabolized, but they can be acetylated by the *lac* operon gene *lacA* (thiogalactoside transacetylase) (Marbach and Bettenbrock 2012). The acetylated derivatives cannot interact with the *lac* repressor anymore and are consequently transported out of the cell. The route of exit is believed to be a concentration gradient (diffusive transport) (Wilson and Kashket 1969). It is believed that catabolite activator protein is essential for expression of the *lac* operon, but not involved in catabolite repression. The main driving force of catabolite repression in the *lac* operon seems to be inducer exclusion, but is still controversial in literature (Crasnier-Mednansky 2008; Görke and Stülke 2008a; Görke and Stülke 2008b).

### Plac system tuning on cellular level is possible using metabolisable inducers

Several authors reported that the p_lac_ system using a non-metabolisable inducer is submitted to all-or-none induction, which impedes tuning on cellular level (Afroz et al. 2014b; Narang and Pilyugin 2008; Novick and Weiner 1957; Ozbudak et al. 2004; Rao and Koirala 2014; Savageau 2011; Siegele and Hu 1997). However, recently, Afroz et al. outlined that the induction characteristics of this system are more complex: tuning on cellular level is possible using lactose as metabolisable inducer (Afroz et al. 2014b), which stands in contrast to the all-or-none response found through TMG and IPTG induction (Marbach and Bettenbrock 2012). The authors concluded that the all-or-none response is due to active transport of IPTG or TMG (Afroz et al. 2014b).

This has the practical implication that high catabolic activity paired with a low transport activity results in a graded response (lactose case), whereas a low catabolic activity paired with a high transport activity yields an all-or-none response (IPTG and TMG case) for the p_lac_ system (Afroz et al. 2014b).

The findings of Afroz et al. (2014b) contradict the observations made by Khlebnikov et al. (2002). Afroz et al. demonstrated a graded response of *E*. *coli* MG1655 upon induction with lactose in a range of 1 to 100 μM. Khlebnikov et al. (2002) observed a bistable response of *E*. *coli* D1210 upon induction with lactose up to 100 μM. This dissent needs to be addressed in further studies.

**Fig. 1:**
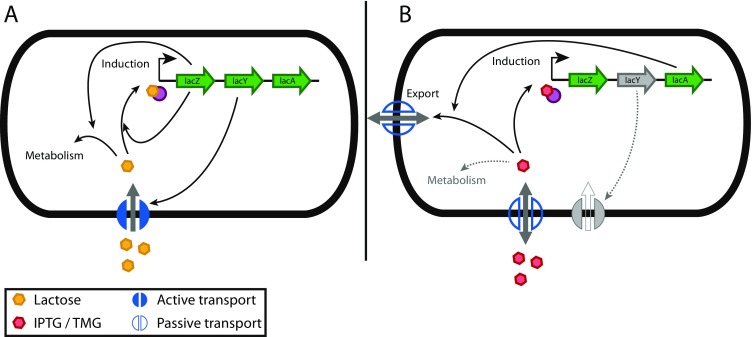
Two possible ways of achieving transcription tuning with the p_lac_ system are described in literature. One reported way to achieve a graded response is to use a *lacZ* ^*+*^ and *lacY* ^*+*^ strain with lactose as inducer (**a**). In this case, lactose induces the expression of *lacZ* (negative feedback) and therefore turns the all-or-none response caused by induction of *lacY* (positive feedback) into a graded response (Afroz et al. 2014b). Another possibility to achieve a graded response is to use a *lacY* ^*−*^ strain with IPTG (**b**) (Jensen et al. 1993; Khlebnikov and Keasling 2002). As the gratuitous inducers (IPTG and TMG) are also recognized by LacY, but cannot be metabolized, the positive feedback needs to be interrupted by deleting *lacY* (Marbach and Bettenbrock 2012). The transacetylase activity of LacA modifies IPTG in a way that it loses its inducing ability and therefore leads to a decrease in single-cell expression levels by decreasing the inducer concentration (Marbach and Bettenbrock 2012). That is why using a *lacA* ^*−*^ mutant is recommended (not shown in this graphic). For better clarity, the role of CRP in the regulation of the lac operon is not illustrated in this graphic, but is nicely described elsewhere (Ozbudak et al. 2004)

As regards the use of lactose as metabolisable inducer, *lacZ* ^*−*^ mutant strain was reported to show all-or-none behaviour when induced by lactose (Afroz et al. 2014b). Hence, for tuning on cellular level in the respective system via lactose as inducer, *lacY* ^*+*^ *lacZ* ^*+*^ strains are obligatory (Fig. 1).

### Plac system tuning on cellular level is possible using lacY mutants

Next to using metabolisable inducers (lactose), tuning on cellular level using the p_lac_ system could be achieved using *lacY* ^−^ mutant strains (lactose transport deficient strains) with gratuitous inducers (Jensen et al. 1993; Khlebnikov and Keasling 2002; Marbach and Bettenbrock 2012) (Fig. 1). Furthermore, Marbach et al. reported that LacA (thiogalactoside transacetylase) activity leads to a decrease in expression levels as the inducer concentration at single-cell level is decreasing (Marbach and Bettenbrock 2012). Therefore, while knockout of *lacY* is mandatory, also *lacA* knockout is recommended when using IPTG as non-metabolisable inducer. A similar behaviour is expected for TMG, however, to our knowledge not proven yet.

### Tuning of the lac operon—application in bioprocesses

Striedner et al. reported transcription of human superoxide dismutase using T7 polymerase under control of lacUV5 in *E*. *coli* K12 strain HMS174(DE3) (intact lac operon) with IPTG (Striedner et al. 2003). In fed-batch experiments with an exponential feed of substrate, IPTG was fed in accordance to the expected biomass at a constant ratio of IPTG to biomass. They were able to maintain the productivity of the culture for a longer period compared to a process with conventional one-point addition of inducer and observed a 3.5-fold increase of overall product titre. However, a proof of tuning on cellular level was not provided. Afroz et al. reported that *E*. *coli* strains with an intact *lac* operon cannot be tuned with IPTG as inducer due to the all-or-none induction phenomenon (Afroz et al. 2014b). Hence, it is to assume that inducer titration using IPTG with intact *lac* operon (as applied in this case) does not result in tuning on cellular level.

In the same study, Striedner et al. used the same host, vector and product, but applied lactose as inducer instead of IPTG (Striedner et al. 2003). In fed-batch experiments with an exponential feed of substrate, lactose was fed as inducer in accordance to a constant specific lactose uptake rate. They were able to maintain the productivity of the culture for a longer period compared to both a process with conventional one-point addition of inducer and the process with IPTG inducer titration and observed twofold increase of overall product titre. In this study, an analytical proof on single-cell level was not provided, but considering the previous discussed findings of Afroz et al. (Afroz et al. 2014b), it is plausible that the increased productivities can be attributed to transcription tuning on single-cell level in this case. In contrast to the process with IPTG, the biomass yield coefficient was constant during the whole process. This might indicate that the cells were not overburdened, since a drop in the biomass yield coefficient is reported to correlate with the metabolic load imposed on the cells (Sagmeister et al. 2012).

Several other studies used *lacY* deficient strains with gratuitous inducer to achieve expression tuning (Hartinger et al. 2010; Hillier et al. 2005; Turner et al. 2005). In order to increase the amount of soluble target protein, Hartinger et al. compared a variety of process conditions and host strains including BL21(DE3) Tuner™. Tuner™ is a *lacZY* ^−^ derivative of *E*. *coli* BL21, which is available in various types and allows tunable transcription upon induction with IPTG. The inducer was added as one-point addition, and induction phase was maintained for 3 h. With varying inducer concentrations, they observed a correlation between IPTG concentration and titre, but no influence on solubility of the product (Hartinger et al. 2010).

Hillier et al. developed a 300-L GMP fermentation process for liver-stage antigen 1 in BL21(DE3) Tuner™ (Hillier et al. 2005). Inducer was added as one-point addition, and induction phase was maintained for 2 h. The tunable ability of the strain was however not exploited as the culture was induced with 1 mM IPTG, which is expected to fully induce the promoter according to other studies (Hartinger et al. 2010; Turner et al. 2005).

Turner et al. used BL21(DE3) Tuner™ to tune the expression of a target protein (cyclomaltodextrinase of thermophilic origin) with respect to solubility (Turner et al. 2005). Cultivations were performed in shake flasks. Induction with IPTG was performed as one-point addition, and induction phases were maintained for 4 h. Inducer concentrations were varied. They observed an influence of transcription rate on the ratio of soluble protein and inclusion bodies. At subsaturating inducer concentrations, higher amounts of soluble protein and activity were detected. With increasing ITPG concentrations, the activity and the amount of soluble protein decreased, whereas the inclusion body concentration increased. These results suggest that tuning of transcription is a useful tool to suppress inclusion body formation.

## araBAD system

### General characteristics of the araBAD system

Another commonly used promoter is the pBAD promoter of the *araBAD* operon. The *araBAD* operon enables *E*. *coli* to take up and metabolize L-arabinose (Schleif 2000). It is induced by L-arabinose. Until now, no gratuitous inducer is reported in literature to induce the wild-type promoter. The non-metabolisable L-arabinose analogue D-fucose acts as inhibitor as it binds to AraC but does not induce transcription (Greenblatt and Schleif 1971; Wilcox 1974). By mutation of *araC*, it is possible to render the system inducable by D-fucose (Beverin et al. 1971). L-arabinose and D-fucose are both transported into the cell by AraE and AraFGH (Daruwalla et al. 1981). The regulation of the *araBAD* operon is excellently described by Schleif (Schleif 2000). It consists of two transporter genes *araE* and *araFGH* (Daruwalla et al. 1981), the genes for arabinose catabolism *araBAD* (Englesberg 1961), a gene of yet unknown function *araJ* (Fritz et al. 2014; Reeder and Schleif 1991) and the regulation gene *araC* (Saviola et al. 1998; Schleif 2010). All genes are under arabinose-inducible control of AraC, which regulates its own expression as well. Besides the positive regulation, it also acts negatively on *araBAD* and *araC*. During the absence of arabinose, AraC acts on *araBAD* as repressor and as activator in the presence of arabinose (Schleif 2010). It is also dependent on the activation by cAMP receptor protein, which renders the system prone to catabolite repression. In the presence of glucose or glucose-6-phosphate, the basal expression level can therefore be held at lower levels (Miyada et al. 1984). AraE and AraFGH do not act independently, and AraE is the more prevailing transporter of these two (Daruwalla et al. 1981).

Transcription kinetics for the system are reported as follows: Transcription from the p_araBAD_ promoter is turned on 1 min after induction with arabinose (Guzman et al. 1995) and turned off in about 3 min after arabinose removal (Fritz et al. 2014). The fast and homogenous shut off of p_araBAD_ transcription is not due to catabolism, but is believed to stand in connection with the arabinose efflux (Fritz et al. 2014).

### araBAD system tuning on cellular level can either be achieved by genetic engineering or process technology

Regarding the all-or-none phenomenon, the system shows formation of subpopulations of producing and non-producing cells at low arabinose concentrations. A graded response is observed when all cells are induced at higher arabinose concentrations (Afroz et al. 2014b). Strains deficient of enzymes for arabinose catabolism are subject to all-or-none induction (Afroz et al. 2014b; Siegele and Hu 1997), and even native strains show a bistable behaviour (Fritz et al. 2014; Makela et al. 2013).

For the araBAD system, it was shown that tuning on cellular level is possible via deleting *araE* and *araFGH* and replacing them by *araE* or *araFGH* under the control of a different promoter (Afroz et al. 2014a; Fritz et al. 2014; Khlebnikov et al. 2000). This is possible, since the positive feedback loop is interrupted.

A graded response of the araBAD operon can also be achieved by process technological means. Using a mixed feed strategy with glucose as main substrate and arabinose as inducing substrate, we demonstrated the tunability of the araBAD operon on cellular level (Sagmeister et al. 2013b).

### Tuning of the araBAD operon—application in bioprocesses

We recently showed that an *E*. *coli* C41 strain can metabolize D-glucose and L-arabinose simultaneously, and that both specific uptake rates can be controlled independently in fed-batch processes, opening the way for a mixed-feed bioprocess for this systems (Sagmeister et al. 2013a). In a subsequent study, the tunability of this mixed-feed process was shown using green fluorescent protein under control of p_araBAD_ as model protein in fed-batch processes (Sagmeister et al. 2013b). Flow cytometry analysis revealed that the culture was tuned on cellular level, although a small subpopulation of non-induced cells was present, but independent of the expression level applied. Afroz et al. did not observe a strictly graded response for the p_araBAD_ system (2014b). Their data revealed that L-arabinose-induced cells (*E*. *coli* MG165) exhibit an all-or-none response at low sugar concentrations and a graded response once all cells have been induced. This observation is in line with our findings and might explain the tunability of p_araBAD_ in a mixed-feed environment as we observed an L-arabinose accumulation for about 10 min upon induction, which was consumed shortly after. This short-term accumulation might lead to the induction of the entire population. We observed a linear relationship between specific arabinose uptake rate (q_s_L-arabinose_) and specific productivity (q_p_). The specific productivity directly responded to adjustments of q_s_L-arabinose_, and within specific ranges, both variables were directly proportional. Hence, an increase in q_s_L-arabinose_ resulted in an increase in q_p_. Within this study, it was demonstrated that tunability of p_araBAD_ in a *E*. *coli* with intact arabinose operon can be achieved by a mixed-feed approach (Sagmeister et al. 2013b).

By constructing arabinose transport deficient strains (*araE-* and *araFGH-*), harbouring a plasmid with a copy of *araE* or *araFGH* under the control of a separate promoter, Khlebnikov et al. were able to generate a dose-dependent and uniform induction on cellular level under subsaturating arabinose concentrations (Khlebnikov et al. 2000). A second plasmid containing green fluorescent protein under the control of the p_araBAD_ promoter allowed examination of single-cell expression levels by flow cytometry analysis. Experiments were performed on millilitre scale. Comparison of different kinds of promoters (p_tac_, p_taclacUV5_ and constitutive promoters of *lactococcus lactis*) and different gene dosages (low- and medium-copy number plasmid, genome integration) for *araE* expression yielded an influence of promoter strength on the homogeneity of induction (Khlebnikov et al. 2001; Khlebnikov et al. 2002). Strong promoters such as the IPTG inducible p_tac_ and the constitutive promoter p_cp18_ resulted in a uniform induction within the culture at arabinose concentrations from 0 to 0.2% weight per volume. Using the weaker p_taclacUV5_, only a part of the culture was induced at low arabinose concentrations. The culture-averaged fluorescence level increased with increasing inducer amount (Khlebnikov et al. 2002).

With their studies, Khlebnikov et al. proved that tunable transcription with p_araBAD_ is possible when the genes encoding for arabinose transport are under control of a separate promoter. Exploitation of this system for development of a bioprocess for recombinant protein production is not yet described in literature.

## rhaBAD system

### General characteristics of the rhaBAD system

Other promoters used for recombinant protein production are the p_rhaBAD_ and the p_rhaT_ promoter of the rha regulon. The *rhaBAD* regulon enables *E*. *coli* to metabolize L-rhamnose. It consists of a rhamnose transporter gene *rhaT* (Muiry et al. 1993; Tate et al. 1992), the genes for rhamnose catabolism *rhaBAD* (Egan and Schleif 1993) and the regulation genes *rhaR* and *rhaS* (Tobin and Schleif 1987; Tobin and Schleif 1990a; Tobin and Schleif 1990b; Vía et al. 1996; Wickstrum et al. 2009). The regulon is controlled by an induction cascade, which is triggered by L-rhamnose. In the presence of rhamnose, RhaR acts as inducer of *rhaS*, which itself induces transcription of *rhaBAD* and *rhaT* (Egan and Schleif 1993; Vía et al. 1996). Cyclic AMP receptor protein (CRP) functions as coactivator for the transcription of all three operons *rhaBAD*, *rhaT* and *rhaS*, which render the system susceptible to catabolite repression (Holcroft and Egan 2000a; Holcroft and Egan 2000b; Wickstrum et al. 2005). RhaS itself is capable of activating *rhaSR* transcription, but due to a lower CRP contribution, it results in a lower transcription than by activation with RhaR. Due to differences in the Shine-Dalgarno sequences, RhaS is expressed at a higher level than RhaR. It therefore leads to a kind of negative autoregulation which results in a decrease of *rhaSR* transcription (Wickstrum et al. 2009).

### Induction repression ratios in the rhaBAD system

In a comparative study, the basal level of expression of p_rhaBAD_ was ten times lower than p_araBAD_ (Haldimann et al. 1998). Due to catabolite repression, it is possible to minimize the basal level of expression (Giacalone et al. 2006; Haldimann et al. 1998). However, on addition of rhamnose to cells growing in the presence of high glucose concentration, the induction levels are comparable to cells growing in the absence of glucose. This suggests that the system is still inducible when being catabolite repressed (Giacalone et al. 2006).

### rhaBAD operon—offering various tunable promoters?

The rha regulon exhibits a strict all-or-none induction; hence, tuning on cellular level is not possible by one-point addition of inducer (Afroz et al. 2014b; Ozbudak et al. 2004; Rao and Koirala 2014). Using an expression system based on the *rhaTRS* locus (p_rhaT_), Giacalone et al. observed a dose-dependent induction on cellular level (Giacalone et al. 2006). Wagner et al. used an expression system based on the p_rhaBAD_ promoter. To the authors’ knowledge so far, no studies were conducted aiming at the knockout of transport proteins to achieve tunable expression using the rhaBAD system.

### Tuning of the rhaBAD operon—application in bioprocesses

Giacalone et al. investigated the tunability of TphoA (PhoA with the toxR transmembrane domain) and green fluorescent protein expression from the *rhaT* promoter (p_rhaT_). Different vectors (low-, medium- and high-copy plasmids) containing the reporter protein under control of p_rhaT_ and the regulatory genes *rhaR* and *rhaS* were constructed and termed pRHA. *E*. *coli* MG1655 (with complete rhamnose pathway) was grown on millilitre scale and induced with L-rhamnose at varying concentrations. The authors observed a dependency of production level on inducer concentration and plasmid copy number. Flow cytometry analysis revealed that the cellular induction level indeed did vary with inducer concentration, but a fraction of non-induced cells remained and did increase with decreasing inducer concentrations. In our understanding, these results suggest that the culture is tuned on population level, and a fraction of the cells is tuned on cellular level. These ambivalent findings need to be addressed in further studies in order to define whether transcription tuning on cellular level is possible or not in this system.

Wagner et al. constructed a BL21(DE3) derivative strain, termed Lemo21(DE3). Using this strain, the possibility to adjust the T7RNAP levels by coexpression of T7 lysozyme under control of the p_rhaBAD_ promoter is reported (Wagner et al. 2008). This strain harbours two plasmids. One plasmid called pLemo contains the regulatory genes *rhaS* and *rhaR* and a variant of T7 lysozyme (LysY) under control of the p_rhaBAD_ promoter. The second plasmid harbours the gene of interest under control of the p_T7_ promoter. The actual tuning is performed with the p_rhaBAD_ promoter and rhamnose as inducer. The resulting T7 lysozyme concentrations reduce the amount of T7RNAP and consequently the expression level of the target protein under control of p_T7_. This system was used for membrane protein production (Schlegel et al. 2012; Wagner et al. 2008) and recombinant protein production in the periplasm of *E*. *coli* (Schlegel et al. 2013)*.* In shake flasks, Schlegel et al. investigated the influence of different rhamnose concentrations on culture homogeneity after 8 h of induction with ITPG (Schlegel et al. 2012). They observed subpopulations of induced and non-induced cells at 0 μM rhamnose, when lysozyme expression was not induced. At increasing rhamnose concentrations, they observed a decrease of the non-induced fraction. Until at a certain rhamnose concentration, a uniform culture was attained. The authors attributed the non-induced fraction of the culture to outgrowth of segregants. Using the Lemo21(DE3) strain, the authors were able to identify the optimal conditions for membrane protein expression and therefore to achieve higher titres of correctly folded product and a more stable production period (Schlegel et al. 2012).

Since two different promoter systems (p_lacUV5_ and p_rhaBAD_) are used, both have to be considered as possible causes of nonuniformity across the culture. For this reason, the system is highly complex with respect to tunability on cellular level, especially considering the findings of Afroz et al. that uniform transcription with IPTG is only possible in *lacY* deficient strains, and that the rhamnose utilization pathway typically responds in a strictly all-or-none fashion (Afroz et al. 2014b).

## proU operon

### General characteristics of the proU operon

The osmoregulated proU operon is one of three proline transport systems in *E*. *coli* providing the cell with the ability to respond to changes in osmolarity in its environment (osmotic stress). The system was reviewed by Lucht et al. (Lucht and Bremer 1994). The operon encodes three proteins: ProV (May et al. 1989), ProW and ProX (Breed et al. 2001) which regulate the transport of glycine-betaine and other osmoprotectants into the cytoplasm at high osmolarity (Gowrishankar 1985; Stirling et al. 1989). To sustain the inner cell pressure (turgor), *E*. *coli* can import K^+^ ions via several K^+^ transport systems as a response to a change of osmolarity in its environment (Booth 1990; Epstein 1986; Sutherland et al. 1986). The import of K^+^ is accompanied by production of glutamate as counter ion (Measures 1975). At higher osmolarities, the cell replaces K^+^ ions by compounds that do not disturb metabolic activities, so called compatible solutes or osmoprotectants (Booth 1990; Epstein 1986; Sutherland et al. 1986). Induction of proU is assumed to be a mixture of elevated potassium-glutamate levels (Leirmo et al. 1987; Lucht and Bremer 1994), changes in DNA super coiling (Higgins et al. 1988; Lucht and Bremer 1994) and a repression mechanism, that only function at low osmolarities (Lucht and Bremer 1994). Transcription cannot be induced at limiting K^+^ concentration in the medium (Sutherland et al. 1986).

### proU operon—promising candidate for tunable recombinant protein expression

The system is not responsible for the import of its inducer. According to the conclusions of Afroz for other systems, it is therefore not expected to show all-or-none behaviour (Afroz et al. 2014b; Rao and Koirala 2014). It directly responds to changes in osmolarity and expression is maintained as long as the osmotic stress exists (Herbst et al. 1994; Lucht and Bremer 1994; Walawalkar et al. 2013).

### Tuning of the proU operon—application in bioprocesses

Herbst et al. constructed a set of expression vectors (termed pOSEX) containing proV and the target genes under control of the p_proU_ promoter (Herbst et al. 1994). With these pOSEX vectors, the expression of β-galactosidase (LacZ) and a carboxyltransferase (GcdA in *E. coli* MKH13 [*ΔputPA101*, *ΔproP2*, *ΔproU608*], a derivative of *E*. *coli* K-12, was studied. Studies were performed on millilitre scale with varying NaCl concentrations. By SDS-PAGE analysis, the authors observed a correlation of target protein concentration and osmolarity of the growth medium. Higher osmolarities resulted in higher target protein concentrations. To be able to attribute these findings to transcription tuning on cellular level, culture uniformity (Afroz et al. 2014b; Rao and Koirala 2014) needs to be addressed by single-cell analytics in further studies. Whether or not the impact of osmotic stress on overall metabolism limits the applicability of the system needs to be investigated (Cheung et al. 2009; Roth et al. 1985; Walawalkar et al. 2013; Weber et al. 2006). Future investigations will also have to consider the impact of osmotic stress on overall metabolism and cell growth (Cheung et al. 2009; Roth et al. 1985; Walawalkar et al. 2013; Weber et al. 2006).

### pprpB

The prp regulon was first described and studied for *Salmonella typhimurium* (Hammelman et al. 1996). A closely related operon was found in *E*. *coli* with a high identity by genomic sequencing (Blattner 1997) and radioactive labelling experiments (Textor et al. 1997). The tunability of the system was investigated in *E*. *coli* (Lee and Keasling 2005). The regulon enables *E*. *coli* to metabolize propionate and is induced in the presence of propionate. The regulon was intensively investigated by the group of Escalante-Semerena (Grimek et al. 2003; Hammelman et al. 1996; Horswill and Escalante-Semerena 1997; Horswill and Escalante-Semerena 1999a; Horswill and Escalante-Semerena 1999b; Horswill and Escalante-Semerena 2001; Palacios 2004; Palacios and Escalante-Semerena 2000; Tsang et al. 1998). It consists of the two operons, prpR and prpBCDE (Horswill and Escalante-Semerena 1997). Under the control of its own promoter p_prpR_, the prpR operon encodes the transcriptional activator for *pprpB* of the sigma-54 family, which is essential for p_prpB_ transcription (Horswill and Escalante-Semerena 1997; Palacios and Escalante-Semerena 2000). The p_prpR_ promoter is not dependent on propionate but is activated by cAMP receptor protein and therefore believed to be solely controlled by catabolite repression (Lee et al. 2005). *PrpBCDE* encodes most of the enzymes of the 2-methylcitric acid cycle for oxidation of propionate to pyruvate (Brock et al. 2001; Brock et al. 2002; Grimek et al. 2003; Horswill and Escalante-Semerena 1999b; Horswill and Escalante-Semerena 2001; Palacios 2004). The single steps of the cycle are well described by Brock et al. (Brock et al. 2002).

P_prpB_ is not directly addressed by propionate. Propionate is converted to 2-methylcitrate (2-MC) in two consequent steps by PrpE and PrpC (Tsang et al. 1998). In the presence of 2-MC, PrpR initiates transcription of *prpBCDE* (Palacios 2004; Palacios and Escalante-Semerena 2000). Besides of PrpR and its coactivator, the sigma-54 transcription factor and the integration host factor (IHF) are needed for *prpBCDE* transcription (Palacios and Escalante-Semerena 2000). The p_prpB_ promoter itself is directly dependent on the activation by CRP. This renders the system prone to catabolite repression (Lee et al. 2005). As propionate is only the precursor of the actual inducer (2-MC), the system could be induced by endogenous metabolic pathways leading to propionate and propionyl-CoA.One such pathway is responsible for the conversion of succinate to propionate (Haller et al. 2000; Lee and Keasling 2005). By taking a look at the net reaction equation of this pathway (propanoyl-CoA + succinate ↔ propanoate + succinyl-CoA) (Keseler et al. 2012), we conclude that no precursor for p_prpB_ induction can synthesized de novo, at least by this pathway. However, possible alternative routes have to be considered. The cell wall is permeable for propionate (Salmond et al. 1984), and no other active import system has been reported to exist in *E*. *coli* up to now. Intracellular inducer concentration can decrease in course of the process due to metabolization of propionate and dilution due to cell growth.

### prpBCDE operon—tuning on cellular level is possible

Lee et al. observed a graded induction on cellular level (Lee and Keasling 2005) in response to the extracellular propionate concentration. With no traits of autocatalytic functionality within its genome (Carrier and Keasling 1999; Rao and Koirala 2014), the prpBCDE operon is a promising tunable system. However, culture uniformity still needs to be proven by single-cell analytics.

### prpBCDE operon—increasing strength

By using PrpR and p_prpB_ of *S*. *typhimurium* in *E*. *coli*, the expression strength was increased in contrast to the *E*. *coli* inherent analogues (Lee and Keasling 2006a; Lee and Keasling 2008). A threefold higher green fluorescent protein production was observed. However, this increase came along with an increase in basal expression levels (Lee and Keasling 2006a).

### Tuning of the prpBCDE operon—application in bioprocesses

Lee et al. constructed several vectors containing *prpR* (activator protein for p_prpB_) and the target gene under control of the *prpBCDE* promoter (p_prpB_), termed pPro (Lee and Keasling 2005). *E*. *coli* DH10B with pPro vectors harbouring green fluorescent protein as marker protein was grown in millilitre scale. Cultures were induced by one-point addition of propionate at varying propionate concentrations. Culture uniformity was verified by flow cytometry measurements 2 and 6 h after induction. The study revealed that the culture was induced uniformly, and that the GFP expression level is a function of the propionate concentration. In a consequent study, Lee et al. investigated pPro vectors containing *prpR* and the *prpBCDE* promoter from *S*. *typhimurium* (Lee and Keasling 2006a; Lee and Keasling 2006b). Comparison with the *E*. *coli* based pPro vectors revealed a threefold higher GFP expression when using the *Salmonella*-based pPro system. As a second step, the tunability on transcriptional level of the *Salmonella*-based pPro system was confirmed by expressing two plant genes encoding coclaurine *N*-methyltransferase (CMT) and norcoclaurine synthase (NCS) in shake flasks (Lee and Keasling 2008). In 2012, the pPro system was registered for patent approval (Jay D. Keasling 2012).

## Conclusions and outlook

Within this contribution, we provide a comprehensive overview on promoter systems that were used to apply expression tuning with *E*. *coli* and discuss promising candidates. To purposefully apply these systems for the benefits of (1) higher overall productivities, (2) debottlenecking of transport pathways and (3) avoiding protein aggregation, it is necessary to consider promoter system specific constraints.

So far, most of the studies dealing with tuning are proof-of-concept studies, performed in millilitre to shake flask scale with one-point addition of inducer, but offer the necessary verification on single-cell level (Lee and Keasling 2005; Schlegel et al. 2012). In some studies, tunable systems were applied for process optimization of recombinant protein production (Hartinger et al. 2010; Turner et al. 2005) or even large scale production (Hillier et al. 2005).

However, only a few studies using tunable strains apply a suitable process control strategy (i.e. induction strategy) (Sagmeister et al. 2013b; Striedner et al. 2010). As discussed elsewhere, one-point addition of inducer is not suited when applying transcription tuning (Marschall et al. 2015). Yet, it is the most common induction method in the reviewed studies (Giacalone et al. 2006; Hartinger et al. 2010; Herbst et al. 1994; Hillier et al. 2005; Lee and Keasling 2008; Turner et al. 2005).

Some promoter systems were reported to offer tunability (Giacalone et al. 2006; Schlegel et al. 2012), which contradicts the strictly all-or-none response observed by Afroz et al. (Afroz et al. 2014b). These ambivalent findings need to be addressed in further studies. Another promising promoter system was identified, possibly enabling transcription tuning by adjustment of the media salt concentration, but needs further investigation.

A lot of issues concerning transcription tuning have been addressed by the reviewed studies. However, some open questions still remain:

- Contradicting findings for already claimed-to-be tunable systems need to be investigated and clarified.
- Verification of culture homogeneity was not always supplied.
- Application of suitable process control strategies is mandatory in order to proof long-term stability of cell growth and recombinant protein production.
- The reviewed promoter systems only represent a small part of available promoter systems for *E*. *coli*, but to our knowledge, they are the ones that were applied for expression tuning so far. Therefore, a lot of other promoter systems, promising as well with respect to tunability, are still waiting to be explored (Balzer et al. 2013).
- Scale-up-related issues of tunable systems have not been investigated so far. In order to apply transcription tuning in industrial processes, the influence of substrate and inducer gradients on the tunability needs to be addressed, especially when following a mixed-feed approach.

Keeping these points in mind, it will be possible to control transcription on single-cell level and thus to an increase in productivity and product quality.

We suggest focusing on promoter systems using inducers that only act on the promoter controlling the desired recombinant genes, in future studies. Relying on inducers that also act on other parts of the cells metabolism, one might unintentionally not only induce the promoter of interest but also turn other adjusting screws that negatively influence recombinant protein expression.

To put it in a nutshell, we believe that expression tuning is a promising tool for industrial application by enabling culture long-term stability and constant product quality and thus ultimately resulting in higher product titres and more cost-efficient production processes.
